# AI Governance in Health Information Practice: A Practical Framework for HIPAA-Compliant AI Adoption

**DOI:** 10.63116/AH.000000009

**Published:** 2026-11-11

**Authors:** Tri’Sheena M. Stokes, Shannon H. Houser

**Affiliations:** 1 Huntsville Hospital Health System; 2 University of Alabama at Birmingham

**Keywords:** artificial intelligence, HIPAA compliance, health information governance, protected health information, AI governance, health care data privacy

## Abstract

Artificial intelligence (AI) tools are increasingly being integrated into health information workflows, including clinical documentation, coding support, revenue cycle operations, and patient engagement systems. While these technologies offer efficiency and enhanced decision support, their use introduces new privacy, security, and compliance risks when protected health information is entered as input, processed by third-party systems, or incorporated into AI-generated outputs. Existing Health Insurance Portability and Accountability Act (HIPAA) compliance structures do not address the complexities of AI-enabled environments, creating governance gaps that expose health care organizations to regulatory and operational risk. This article presents a practical governance framework that integrates AI oversight into existing HIPAA compliance programs. Drawing on compliance literature, regulatory guidance, and emerging AI governance models, the framework adapts core elements of the Office of Inspector General compliance model to address AI-specific risks. We proposed AI-centered policies and procedures, governance leadership, workforce training, auditing and monitoring, and incident response planning. This article provides implementation guidance tailored to health information practice, including workflow-based examples and phased adoption strategies. By positioning AI governance as an extension of established information governance responsibilities, health information professionals can proactively safeguard patient privacy, strengthen regulatory compliance, and support responsible innovation in AI-enabled health care environments.

## Introduction

Artificial intelligence (AI) is no longer an emerging technology in health care; it is embedded in routine operational workflows. AI-enabled systems now support clinical documentation summarization, computer-assisted coding, predictive analytics, patient communication, and revenue cycle processes.^1^ As these systems increasingly process protected health information (PHI), they expand the risk surface for privacy breaches and compliance failures.^2^ Although the Health Insurance Portability and Accountability Act (HIPAA) establishes safeguards for PHI, many health care organizations lack structured governance models capable of addressing the operational and compliance complexities introduced by AI-driven environments.^3^

Health information (HI) professionals are uniquely positioned to lead this governance transition. The HI discipline has long been responsible for data integrity, privacy protection, and regulatory oversight.^4^ However, AI introduces new data flows, vendor relationships, and automated decision processes that extend beyond traditional information governance frameworks. Without proactive AI governance, organizations face increased risk of unauthorized PHI disclosure, inappropriate vendor data use, and regulatory liability.^2^^,^^5^

The central challenge is no longer whether AI will be adopted in health care, but how it will be governed. Rapid AI adoption has outpaced the development of operational compliance frameworks, creating a governance gap in which innovation advances, while oversight lags.^3^^,^^6^ Addressing this gap requires translating established HIPAA principles into AI-enabled operational settings that account for third-party systems, automated processing, and evolving vendor responsibilities.^5^

This article addresses a practical question relevant to everyday HI practice: How can health care organizations integrate AI governance into existing HIPAA compliance programs? The purpose of this manuscript is to present a structured, implementation-focused framework that enables HI professionals to manage AI-related risk while supporting responsible innovation. The proposed framework provides practical guidance for integrating AI oversight into existing HIPAA compliance programs while supporting responsible adoption of AI-enabled health care technologies.^1^^,^^4^

### AI Risk Landscape in HI Practice

AI technologies are increasingly being integrated into core HI functions, extending beyond pilot initiatives into operational use. AI-enabled tools support computer-assisted coding, document summarization, automated patient communication, clinical decision support, and revenue cycle analytics.^1^^,^^7^ For purposes of this discussion, AI-enhanced systems support human decision-making and workflow processes, AI-driven systems perform substantial automated processing or generate outputs that influence operational activities, and AI-related systems broadly encompass technologies and workflows that incorporate AI capabilities within HI environments. Many of these AI-enabled applications rely on automated data platforms, creating distinct pathways for PHI exposure and oversight challenges beyond those encountered in traditional electronic health record (EHR) systems.

AI introduces privacy and compliance risks throughout the processing, storage, and use of HI. PHI may be entered directly into AI systems as prompts, processed by external vendors, stored in cloud-based infrastructures, or embedded within AI-generated outputs.^2^^,^^5^ In some cases, AI vendors may retain data to improve algorithms or train future models, raising questions about ownership, authorization, and secondary use of PHI.^5^^,^^8^ These practices challenge conventional interpretations of business associate responsibility and blur accountability boundaries between health care organizations and technology developers.

Operational risk is further amplified by workforce behavior and the complexity of AI-enabled systems. Employees may unknowingly expose PHI by using unauthorized public AI tools or by misunderstanding how vendor systems store and reuse data.^2^^,^^9^ Unlike traditional HI systems, AI platforms may operate outside established audit controls, increasing the likelihood of undetected disclosure. The combination of automated processing and opaque vendor practices creates an environment in which privacy violations may occur without immediate visibility.

The consequences of AI-related compliance failures extend beyond regulatory penalties. Health care data breaches have been steadily increasing, with hacking and information technology incidents representing the predominant cause of large-scale PHI exposure.^10^ AI adoption expands the digital attack surface by introducing additional access points, third-party integrations, and automated data flows. A breach affecting AI systems can disrupt operations, delay care delivery, generate financial losses, and erode patient trust.^10^^,^^11^

Patients bear the greatest long-term privacy and security risks associated with AI-related breaches of PHI. Compromised PHI can result in identity theft, fraudulent billing, and loss of confidence in health care institutions.^11^ AI-driven systems that aggregate and process data from multiple sources may increase the scope of information exposed when a breach occurs, particularly when large volumes of PHI are processed within a single AI environment. As health care increasingly relies on data-driven technologies, maintaining patient trust becomes inseparable from maintaining strong governance.

These risks demonstrate that AI governance is not an optional innovation strategy; it is a core extension of HI compliance. Because AI systems continue to evolve more rapidly than regulatory safeguards, deliberate oversight is essential to protect PHI. Effective governance requires recognizing AI not as a standalone technology, but as an integrated component of the HI ecosystem that must be managed with the same rigor applied to traditional data systems.

### Regulatory and Governance Context

HIPAA remains the primary regulatory framework governing the protection of PHI. However, HIPAA was developed in an era preceding modern AI ecosystems and does not explicitly address many operational realities introduced by automated data processing, large language models, and vendor-hosted AI platforms.^3^^,^^5^ As health care organizations integrate AI into routine workflows, emerging AI technologies raise new regulatory and governance considerations regarding developer obligations, vendor accountability, and responsibility for AI-generated outputs that contain PHI.^5^

Regulatory agencies increasingly recognize the need to address AI-related privacy and security risks. The Federal Trade Commission has emphasized that organizations handling consumer HI must avoid unfair or deceptive data practices, including undisclosed secondary data use and inadequate protection measures.^12^ These expectations extend to AI systems that process health data, particularly when vendors operate outside traditional covered entity classifications. Similarly, the National Institute of Standards and Technology introduced the Artificial Intelligence Risk Management Framework to promote trustworthy AI development and deployment.^13^ Although voluntary, this framework signals a growing federal expectation that organizations proactively manage AI risk rather than rely solely on reactive enforcement.

Health care–specific governance models are also evolving. The Health Information Trust Alliance has incorporated AI considerations into its cybersecurity certification framework, encouraging organizations to align privacy-preserving technologies with established HIPAA security controls.^9^ Emerging international policies, including the European Union’s Artificial Intelligence Act, further highlight global recognition that AI requires structured governance to protect personal data.^14^ While these policies differ in scope and jurisdiction, they collectively demonstrate a shift toward formal oversight of automated systems that process sensitive information.

Despite increasing regulatory attention, external guidance alone cannot close the operational governance gap. Health care organizations remain responsible for translating regulatory expectations into internal compliance practices.^3^^,^^10^ Effective AI governance must therefore be integrated into existing HIPAA compliance infrastructures rather than treated as a separate regulatory initiative. Embedding AI oversight within established compliance programs allows organizations to adapt familiar frameworks to new technological realities while maintaining continuity with long-standing privacy protections.

## AI Governance Framework

### Framework Overview: Five-Element Model

To address the governance gap created by rapid AI adoption and existing compliance infrastructure, health care organizations require an operational model that operationalizes existing regulatory frameworks into AI-enabled environments. Rather than constructing an entirely new compliance architecture, this manuscript proposes leveraging established HI governance principles to manage AI-specific risk. This approach aligns AI oversight with longstanding HIPAA compliance practices while acknowledging the distinctive characteristics of automated and data-driven systems.^15^

The proposed framework draws from the Office of Inspector General (OIG) model of an effective compliance program, a structure widely recognized across health care for promoting ethical operations and regulatory adherence.^15^^,^^16^ The OIG model emphasizes written policies and procedures, designated leadership, workforce education, monitoring and auditing, and corrective action—core elements that remain directly applicable in AI contexts. However, AI systems introduce additional considerations, including vendor transparency, algorithmic accountability, model performance oversight, and large-scale automated processing of PHI. Integrating AI governance into existing compliance structures allows organizations to build upon familiar processes rather than creating parallel or fragmented oversight mechanisms.

The framework is also informed by established information governance principles that emphasize accountability, stewardship, transparency, and oversight of HI assets. These principles support a structured approach to managing AI-related risks while ensuring that organizational responsibilities for privacy, security, and regulatory compliance remain aligned with broader information governance objectives.

Accordingly, this manuscript adapts the OIG compliance framework into the following 5 AI-specific governance elements: (1) AI-centered policies and procedures, (2) AI governance leadership, (3) workforce AI compliance training, (4) AI auditing and monitoring, and (5) AI incident response and corrective action. These elements translate regulatory expectations into structured operational practices that HI professionals can implement within existing organizational infrastructures. The framework emphasizes practical integration rather than theoretical compliance, supporting consistent oversight of AI-enabled workflows.

Positioning AI governance as an extension of HI governance reinforces the central role of HI leadership in protecting PHI. AI systems should be governed with the same rigor applied to EHRs, revenue cycle systems, and other core data infrastructures. By embedding AI oversight within HIPAA compliance infrastructure and established information governance structures, health care organizations can reduce regulatory uncertainty, strengthen accountability, and support responsible technological innovation.^10^^,^^15^ This framework contributes to the emerging body of AI governance literature by translating regulatory principles into actionable guidance tailored to HI practice.

The five-element AI governance framework (Figure 1) provides a structured and implementation-oriented model for operationalizing AI oversight within existing HIPAA compliance and HI governance systems.

**Figure 1.**
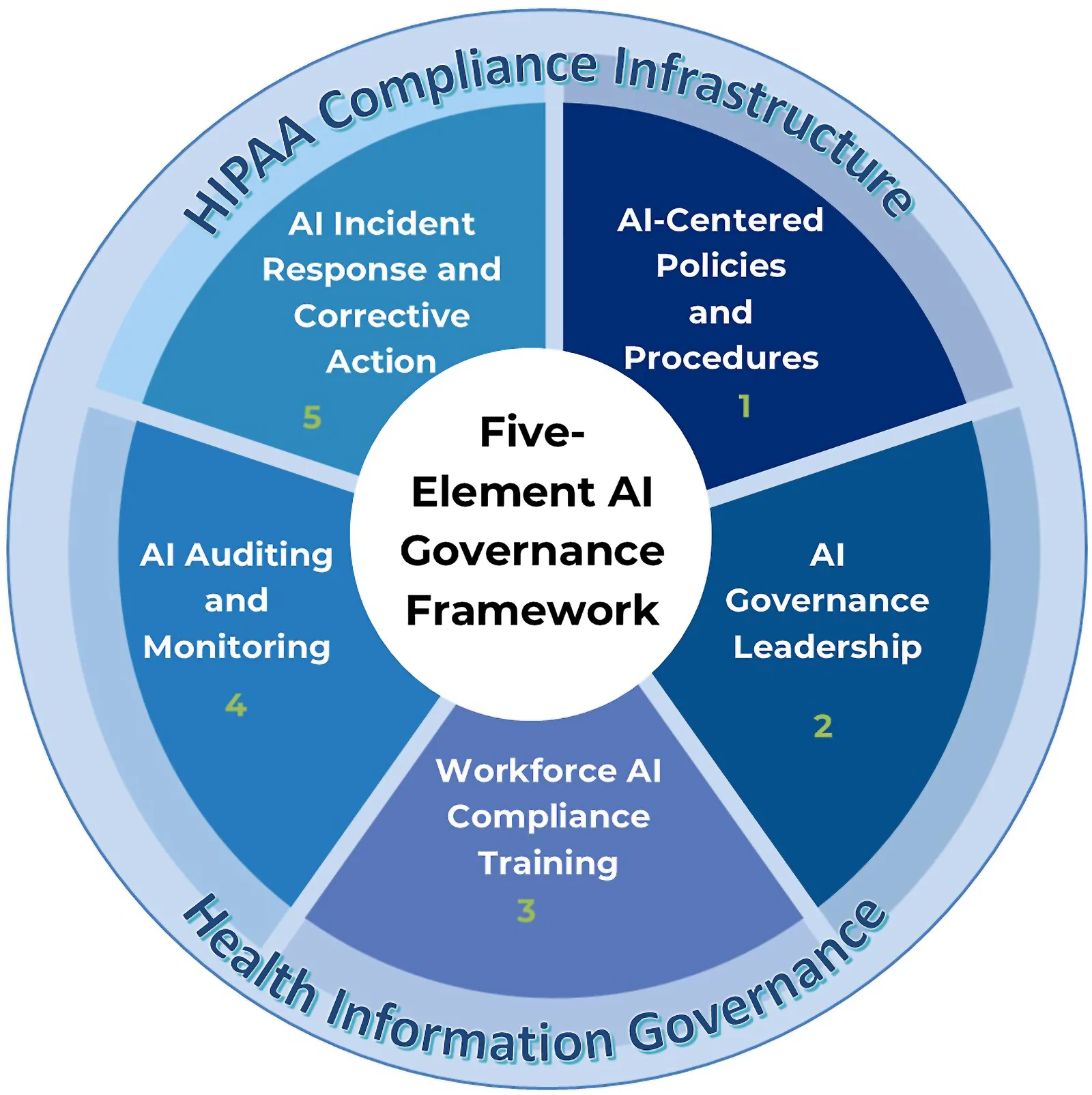
Five-Element AI Governance Framework. Conceptual model integrating AI oversight into Health Insurance Portability and Accountability Act compliance infrastructure and health information governance through the following 5 operational domains: AI-centered policies and procedures, AI governance leadership, workforce AI compliance training, AI auditing and monitoring, and AI incident response and corrective action. AI, artificial intelligence.

### The Five Elements of the AI Governance Framework

The Five-Element AI Governance Framework translates established compliance principles into operational practices tailored to AI-enabled environments. Each element represents a distinct domain of governance oversight while functioning as part of a continuous compliance cycle. Together, these elements provide HI professionals with a structured approach to managing AI-related risk within existing organizational infrastructures.

#### AI-Centered Policies and Procedures

AI governance begins with written policies and procedures that explicitly address AI-enabled technologies. Traditional HIPAA compliance policies typically focus on EHRs, access controls, and breach response. However, AI systems introduce new operational variables, including automated data processing, model retraining, third-party vendor involvement, and algorithm-generated outputs containing PHI.

AI-centered policies should define acceptable AI use, establish system approval and review processes, clarify documentation standards for AI-generated content, and specify data-sharing limitations with vendors. For example, an HI department implementing AI-assisted coding software should establish verification protocols requiring certified coders to validate AI-generated code suggestions before claim submission. Vendor contracts should explicitly prohibit secondary data use for model training without organizational authorization, and formalizing these requirements within compliance documentation strengthens regulatory defensibility while promoting consistent operational standards.^15^^,^^16^

#### AI Governance Leadership

Effective AI oversight requires clearly designated governance leadership. Responsibility for AI-enabled systems should not be diffused across departments without defined accountability. HI leaders, compliance officers, privacy officers, and information technology executives should collaborate with other relevant stakeholders, as appropriate, to establish a structured governance model to oversee procurement, deployment, monitoring, and retirement of AI systems. For instance, before deploying an AI-powered clinical documentation summarization tool, an HI governance committee may conduct a structured review assessing data inputs, storage locations, audit log capabilities, and potential documentation integrity risks. Leadership oversight ensures that implementation decisions consider both operational efficiency and regulatory implications.

Formal governance committees or advisory groups provide multidisciplinary review and ongoing oversight. Clear accountability mechanisms ensure that AI-related decisions align with organizational compliance standards and privacy protections.

#### Workforce AI Compliance Training

Workforce education is essential to maintaining AI-related compliance. Even well-designed governance policies can fail if employees misunderstand how AI systems process, store, or transmit PHI. Staff may inadvertently expose sensitive information through unauthorized AI tools or overreliance on automated outputs.

AI compliance training should address appropriate system use, verification of AI-generated documentation, recognition of system limitations, and procedures for reporting suspected errors or privacy concerns. For example, HI staff should receive instruction on reviewing AI-generated discharge summaries for documentation completeness and compliance before physician validation and confirming that PHI is not entered into unauthorized public AI platforms. Training should emphasize that AI augments professional judgment rather than replaces it. Integrating AI-specific content into existing HIPAA training programs ensures continuity with established compliance culture while preparing staff for evolving digital workflows.

#### AI Auditing and Monitoring

Continuous auditing and monitoring are critical in AI-enabled environments where automated systems may process PHI at scale with minimal direct human intervention. AI auditing should include periodic review of system outputs for accuracy and bias, validation of data sources, assessment of algorithm performance before implementation and over time, and access log monitoring. As a practical example, HI departments may conduct quarterly audits of AI-assisted coding variance rates or review documentation discrepancies between AI-generated summaries and final provider notes. Monitoring vendor system updates and maintaining version-control documentation can help detect performance drift or changes in data handling practices.

Incorporating AI systems into established audit protocols reinforces accountability and strengthens regulatory defensibility. Without structured monitoring, AI-related compliance risks may remain undetected until significant harm occurs.

#### AI Incident Response and Corrective Action

Despite preventive safeguards, AI-related compliance incidents may occur. Effective governance therefore requires structured incident response procedures integrated into existing breach notification and corrective action protocols.

For example, if an AI vendor inadvertently stores PHI in an unsecured environment or an employee enters identifiable patient data into a nonapproved generative AI platform, escalation procedures should align with established HIPAA breach evaluation protocols. Response actions may include vendor notification, forensic investigation, workforce retraining, temporary suspension of AI functionality, and documentation of corrective measures.

Embedding AI-specific considerations into existing response frameworks reduces ambiguity during high-risk events and supports timely mitigation of privacy and compliance failures.

## Implications for HI Practice

AI governance represents a significant evolution in HI practice rather than a peripheral technology initiative. As AI systems become integral to documentation, coding, revenue cycle operations, and analytics platforms, oversight responsibilities naturally extend to HI professionals who have long served as stewards of data integrity and regulatory compliance. The five-element AI governance framework positions HI leaders as not merely participants in AI adoption but architects of structured and accountable oversight.

First, AI governance expands the scope of traditional information governance competencies. This shift reflects a broader transformation within the HI profession, from managing data assets to identifying, managing, and mitigating digital risks associated with AI-enabled technologies. HI professionals must understand not only HIPAA safeguards but also vendor data practices, algorithmic outputs, automated decision pathways, and system-level implications for privacy and documentation integrity. Effective oversight therefore requires closer collaboration with information technology, compliance, and executive leadership teams. Positioned at the intersection of operational data management and regulatory accountability, HI departments are well equipped to guide responsible AI deployment.^17^

Second, workforce preparation must become a strategic priority.^17^ HI professionals require advanced competencies in validation, oversight, and audit interpretation of AI-generated outputs to ensure documentation accuracy and regulatory compliance. Educational curricula and continuing professional development programs should incorporate AI literacy, governance principles, and digital risk management strategies to equip practitioners with the skills necessary for effective oversight.^17^ Embedding AI governance competencies within professional standards reinforces HI leadership in safeguarding data integrity in increasingly technology-driven environments.

Third, structured AI governance enhances organizational resilience. Health care organizations that proactively integrate AI oversight into existing HIPAA compliance programs may be better positioned to respond to regulatory scrutiny, vendor disputes, and emerging privacy risks.^13^ Rather than reacting to incidents, organizations can demonstrate deliberate and documented governance processes that reflect a mature compliance culture.

Finally, integrating AI governance within HI practice sustains patient trust.^18^ As health care systems rely more heavily on data-driven technologies, public confidence depends on transparent oversight and responsible stewardship of PHI. HI professionals play a central role in maintaining that trust by ensuring technological advancement remains aligned with privacy protections and documentation integrity.

As health care continues to evolve within complex digital ecosystems, sustained governance will be essential. Through structured, profession-centered oversight frameworks, HI leaders can guide organizations through technological transformation while preserving the foundational principles of privacy, security, and data integrity that define the discipline.

## Conclusions

The accelerating integration of AI into health care operations underscores the need for structured governance within existing compliance systems. While regulatory frameworks such as the HIPAA establish foundational privacy and security protections, effective oversight of AI-enabled technologies requires deliberate operational integration rather than reactive adaptation.

The five-element AI governance framework offers a structured, implementation-oriented approach for translating established compliance principles into actionable oversight practices tailored to AI-driven environments. By embedding AI-centered policies, defined governance leadership, workforce training, auditing and monitoring processes, and incident response protocols within established HI governance structures, organizations can strengthen accountability while maintaining continuity with longstanding regulatory safeguards.

As AI adoption continues to expand across health care settings, future research should evaluate implementation of the proposed framework in diverse organizational environments and assess its effectiveness in supporting AI governance, regulatory compliance, and risk management outcomes. Such efforts may further refine the framework and provide evidence to guide its practical application across evolving HI ecosystems.

For HI professionals, AI governance represents a natural extension of core responsibilities in data stewardship, documentation integrity, and privacy protection. As digital health ecosystems continue to evolve, sustained leadership from the HI profession will be essential to ensure that technological advancement remains aligned with ethical standards, regulatory expectations, and patient trust.

## Author Contributions

Stokes conceptualized the original white paper and framework. Houser contributed to manuscript development, organization, and refinement for publication. Both authors contributed to writing, review, and final approval of the manuscript.

## Conflicts of Interest

The authors declare no conflicts of interest.

## Disclosures

The authors have nothing to disclose.

## Funding

The authors received no funding for this research.

CE Quiz

## References

[1] Ali O, Abdelbaki W, Shrestha A, Elbasi E, Alryalat MAA, Dwivedi YK. A systematic literature review of artificial intelligence in the healthcare sector: benefits, challenges, methodologies, and functionalities. J Innov Knowl. 2023;8(1):100333. doi:10.1016/j.jik.2023.100333.

[2] Marks M, Haupt CE. AI chatbots, health privacy, and challenges to HIPAA compliance. JAMA. 2023;330(4):309-310. doi:10.1001/jama.2023.9458. 37410450

[3] Alder S. HIPAA history. HIPAA Journal. Updated January 2, 2026. Accessed August 7, 2026. https://www.hipaajournal.com/hipaa-history/.

[4] American Health Information Management Association. AHIMA public policy statement: data quality and integrity. Published 2022. Accessed August 14, 2026. https://www.ahima.org/advocacy/policy-statements/data-quality-and-integrity/.

[5] Rezaeikhonakdar D. AI chatbots and challenges of HIPAA compliance for AI developers and vendors. J Law Med Ethics. 2023;51(4):988-995. doi:10.1017/jme.2024.15. PMC10937180 38477276

[6] Privacy International. Large language models and data protection. Published August 14, 2024. Accessed August 14, 2026. https://privacyinternational.org/explainer/5353/large-language-models-and-data-protection.

[7] Kaul V, Enslin S, Gross SA. History of artificial intelligence in medicine. Gastrointest Endosc. 2020;92(4):807-812. doi:10.1016/j.gie.2020.06.040. 32565184

[8] Hansen J, Carlin K, Hart A, Johnson J, Wells S, Kiel JM. Updating HIPAA security to respond to artificial intelligence. J AHIMA. Published November 13, 2023. Accessed August 14, 2026. https://journal.ahima.org/page/updating-hipaa-security-to-respond-to-artificial-intelligence.

[9] Basil NN, Ambe S, Ekhator C, Fonkem E. Health records database and inherent security concerns: a review of the literature. Cureus. 2022;14(10):e30168. doi:10.7759/cureus.30168. PMC9647912 36397924

[10] Alder S. Healthcare data breach statistics. HIPAA Journal. Published June 18, 2026. Accessed August 14, 2026. https://www.hipaajournal.com/healthcare-data-breach-statistics/.

[11] Looi JCL, Allison S, Bastiampillai T, Maguire PA, Kisely S, Looi RH. Mitigating the consequences of electronic health record data breaches for patients and healthcare workers. Aust Health Rev. 2024;48(1):4-7. doi:10.1071/AH23258. 38109886

[12] Federal Trade Commission. FTC proposes amendments to strengthen and modernize the Health Breach Notification Rule. Published May 18, 2023. Accessed August 14, 2026. https://www.ftc.gov/news-events/news/press-releases/2023/05/ftc-proposes-amendments-strengthen-modernize-health-breach-notification-rule.

[13] Tabassi E. *Artificial Intelligence Risk Management Framework (AI RMF 1.0).* National Institute of Standards and Technology; 2023 NIST AI 100-1. doi:10.6028/NIST.AI.100-1.

[14] European Parliament. Artificial Intelligence Act: MEPs adopt landmark law [press release]. Published March 13, 2024. Accessed November 29, 2024. https://www.europarl.europa.eu/news/en/press-room/20240308IPR19015/artificial-intelligence-act-meps-adopt-landmark-law.

[15] US Department of Health and Human Services, Office of Inspector General (OIG). General Compliance Program Guidance. Published November 6, 2023. Accessed March 20, 2026. https://oig.hhs.gov/documents/compliance-guidance/1135/HHS-OIG-GCPG-2023.pdf.

[16] Health Care Compliance Association. *Complete Healthcare Compliance Manual 2024.* Society of Corporate Compliance and Ethics; 2024. Accessed August 14, 2026. https://books.google.com/books/edition/Complete_Healthcare_Compliance_Manual_20/SWFw0AEACAAJ.

[17] Houser SH, Crew D, Flite CA, Goethals C. Shaping the future of the health information profession: the impact of emerging technologies on the workforce. Adv Health Inf Sci Pract. 2025;1(2):RBQG5825. doi:10.63116/RBQG5825. PMC12348412 40979236

[18] Bracic A, Spector-Bagdady K, Towle S, Zhang R, James CA, Price WN II. Factors for patient trust and acceptance of medical artificial intelligence. JAMA Netw Open. 2026;9(3):e260815. doi:10.1001/jamanetworkopen.2026.0815. PMC12964161 41784960

